# An Estimate of Severe and Chronic Fungal Diseases in the Republic of Kazakhstan

**DOI:** 10.3390/jof4010034

**Published:** 2018-03-08

**Authors:** Vadim M. Kemaykin, Nariman B. Tabinbaev, Mahira S. Khudaibergenova, Anastasia A. Olifirovich, Layzzat M. Abdrakhmanova, David W. Denning, Nikolai Klimko

**Affiliations:** 1 National Scientific Center for Oncology and Transplantology, Astana 020000, Kazakhstan; hematology.astana@gmail.com (V.M.K.); nariman_tab@mail.ru (N.B.T.); mahira68@mail.ru (M.S.K.); nastas-ja@bk.ru (A.A.O.); layzzat_uprav18@mail.ru (L.M.A.); 2 The National Aspergillosis Centre, Wythenshave Hospital, The University of Manchester, Manchester Academic Health Science Centre, Manchester M23 9LT, UK; ddenning@manchester.ac.uk; 3 Leading International Fungal Education (LIFE), Cheshire SK10 9AR, UK; 4 Department of Clinical Mycology, Allergy and Immunology, I. Metchnikov North Western State Medical University, St. Petersburg 191015, Russia

**Keywords:** Leading International Fungal Education (LIFE) Program, mycoses, fungal diseases, Republic of Kazakhstan

## Abstract

Our work aimed to generate a preliminary estimation of severe and chronic fungal diseases in the Republic of Kazakhstan with a model proposed by LIFE (Leading International Fungal Education). Calculations were carried out on data from 2015. Published results of studies of mycoses in Kazakhstan were identified; in the absence of national data from the scientific literature, the frequency of life-threatening and serious mycoses in defined groups of patients at risk from other countries were taken into account. We also used analogous estimations of mycoses in the Russian Federation. We estimate that 300,824 patients (1.7% of the population) were affected by severe and chronic mycotic diseases. There were an estimated 15,172 cases of acute mycoses, notably tinea capitis in children (11,847), *Pneumocystis* pneumonia and invasive candidiasis, and 285,652 of chronic fungal diseases. The most frequent were chronic recurrent vulvovaginal candidiasis (273,258 cases) and chronic pulmonary aspergillosis (6231). There is uncertainty about the prevalence of asthma in adults; the official number is 12,478 patients, but a prevalence estimate of 1.47% from a WHO consortium yields a prevalence of ~170,000 affected. We have used the official figures to generate the prevalence of fungal asthma, but it is likely to be a significant underestimate. Conclusion: Results of investigation indicate significant prevalence of severe and chronic mycoses in the Republic of Kazakhstan.

## 1. Introduction

Mycoses have been increasing in recent years as a result of better recognition and increasing numbers of immunocompromised patients [1,2,3]. Although antifungal prophylaxis is commonly used in leukemia and transplant patients, the majority of patients at risk do not receive antifungal treatment until a diagnosis is made or highly suspected. Many diagnostic and therapeutic advances have been made in the last 15 years. From a public health perspective, an estimate of the burden of infection, its health economic consequences, and contributions to morbidity and mortality are critical to a rounded assessment of the most pressing needs of different patient groups and priority setting. Such an estimation has never been previously attempted for the Republic of Kazakhstan.

To compare and coordinate epidemiological data and link it with health professional education, the Fungal Infection Trust set up LIFE (Leading International Fungal Education) in 2012 [4]. Some mycotic diseases cannot be estimated from underlying disease and risk, such as tinea capitis and fungal keratitis, and so estimates of these infections tend to reflect incidence or prevalence in one or a small number of localities, whereas infection rates in, for example, transplant recipients, can be estimated with a much higher degree of precision. Using this approach, we evaluated the burden of serious mycoses in Kazakhstan.

## 2. Materials and Methods

The calculations by the LIFE modeling approach were performed using indicators and data from 2015. Published data on mycoses in Kazakhstan were studied. In the absence of official data, we estimated the size of each at risk group and then assessed the national prevalence and morbidity rates using data from the literature. Similar approaches used in the Russian Federation guided our estimates [5].

Statistical data about number and composition of the population of Kazakhstan were obtained from the statistics of the Ministry of Health and Social Development (MHSD), 2015 [6,7].

The evaluation of the morbidity due to scalp mycoses was based on data from the MHSD, 2015 [6].

The number of women of reproductive age (15–50 years) in Kazakhstan in 2015 accounted for 4,554,300 [6]. The number of patients with recurrent vulvovaginal candidiasis (rVVC) was calculated using anonymized, randomly collected data from international epidemiological investigations showing that this disease occurs in 6% of women of reproductive age [8].

Information on the number of patients living with HIV/AIDS in Kazakhstan was received from the report of the MHSD, 2015—103 per 100,000 population [7]. Oropharyngeal candidiasis has been identified in 90% of HIV-infected patients with CD4^+^ < 0.2 × 10^9^/L + 90% of HIV/AIDS patients not taking antiretroviral therapy ART and candidal oesophagitis—in 20% of HIV-infected patients with CD4^+^ < 0.2 × 10^9^/L + 20% of HIV/AIDS patients are not on anti-retroviral therapy, and 0.5% if on ART [9].

According to Klimko et al. in Russia [5], the frequency of candidemia and *Candida* peritonitis accounted for 0.37 per 1000 hospitalized patients, and this figure was applied. The total number of hospitalized patients in 2015 was obtained from data of the MHSD [6].

The estimation of hematological diseases was guided by the data of the MHSD, 2015—4335 per 100,000 population [6]. The risk of the development of invasive aspergillosis (IA) in patients with hematological diseases was calculated as described elsewhere [5,10,11]. The frequency of mucormycosis development in the general population was calculated using data of prevalence of acute myeloid leukemia in the report of the MHSD, 2015, supplemented by general population data. The estimation of chronic obstructive pulmonary disease COPD prevalence was guided by statistical data [6].

Data on the annual incidence of pulmonary tuberculosis were received from the reports of the MHSD [6]. The annual incidence of chronic pulmonary aspergillosis (CPA) was estimated as described previously [5,12]; the annual number of cases of pulmonary tuberculosis with cavities left after treatment (≈12%) had a 22% risk of CPA in addition to a 2% risk in the remainder. As tuberculosis incidence is not very high in Kazakhstan, it was assumed that other underlying conditions associated with CPA accounted for two-thirds of the cases [13].

The prospective number of patients with allergic bronchopulmonary aspergillosis (ABPA) was calculated by applying a general rate of 2.5% of the number of adults with bronchial asthma (BA) in addition to 15% of the adult patients with cystic fibrosis (CF) [14]. The number of patients with asthma was derived from the reports of the MHSD [6]. For estimation of the patients with severe asthma with fungal sensitization (SAFS), it was assumed that 10% of patients with asthma had a severe clinical course and 33% were sensitized to one or more fungi [15]. Data about ABPA frequency in CF patients were taken from the scientific literature, ~15% in adults [16]. About 21% of all patients with CF are adults [5].

To account for the morbidity from and burden of cryptococcal meningitis we used Russian data; cryptococcal meningoencephalitis developed in 0.44% patients with HIV-infection [5]. The number of cases of *Pneumocystis* pneumonia (PCP) was determined by assuming the risk extends over 2 years and 60% of HIV-infected patients with CD4^+^ < 0.2 × 10^9^/L develop PCP [17,18].

## 3. Results and Discussion

The population of the Republic of Kazakhstan in 2015 was 17,670,600 people [6]. The adults comprised 65.3% of the population, women—51.8%. The estimated gross domestic product per capita was USD $10,616 in 2015 [19]. The estimates of fungal disease incidence and prevalence in the Republic of Kazakhstan are shown in Table 1.

### 3.1. Candidiasis of Mucous Membranes

Recurrent VVC is the most common mycotic disease in women. It is characterized by four or more recurrences annually [4]. The calculations showed that in 2015 in the Republic, 273,258 women suffered from this disease, which accounts for approximately 2985 cases per 100,000 females. These data are similar to those of the Russian Federation (3481/100,000) [5] and Uzbekistan (3339/100,000) [20], and higher than in Ukraine (1961/100,000) [21].

Kazakhstan in 2015 registered 2366 new cases of HIV (CD4^+^ < 0.2 × 10^9^/L − 17.8%) and 17,726 living with HIV patients which accounts for 103 persons per 100,000 population. Of these, 74.6% received ART [7]. Consequently, the number of HIV-infected patients with oropharyngeal candidiasis was 4393 (24.8 cases per 100,000 population), with oesophageal candidiasis—1042 (5.9/100,000). The numbers affected are higher than incidence in Uzbekistan (16.09 and 6.72 respectively), but lower than incidence in the Russian Federation (42.4 and 9.42 respectively) [5,20]. In Ukraine the prevalence of oesophageal candidiasis is probably much higher (30/100,000) [21].

### 3.2. Mycosis of the Scalp

According to the data of the MHSD in 2015, the total number of affected patients with tinea capitis was 11,847 or 66.9/100,000 [6]. The numbers affected are higher than incidence in the Russian Federation (42.6/100,000) [5] and Uzbekistan (23.8/100,000) [20], but much higher than incidence in European countries [18].

### 3.3. Invasive Candidiasis

The number of patients hospitalized in Kazakhstan in 2015 was 2,550,816 persons [6]. Among these patients, the total number of patients with candidaemia and intra-abdominal candidiasis in this period was 765 (4.3 cases per 100,000 population). These data are lower than those seen in the Russian Federation (8.29/100,000 population), and correspond to Uzbekistan, Ukrainian and mean European parameters [5,18,20,21].

### 3.4. Aspergillosis of the Respiratory Tract

Hematological diseases, in particular AML, are an important risk factor for development of IA. According to the mean European parameters, the risk of IA occurrence in this category of patients is equal to approximately 10% [18]. According to the data of the MHSD in the Republic, 113 patients developed AML in 2015, and IA was diagnosed in 11 (10%) of them. Taking into account that there were no organ transplantation procedures, the annual incidence of IA in hematological malignancy was calculated from the AML cohort as by 50% of all IA cases are in this group [18,22]. Lung cancer was diagnosed in 4684 patients in 2012 [23] and, with a rate of IA of 2.6% [24], another 121 patients with IA are likely. In addition, 1.3% patients with COPD who were hospitalized were assumed to have IA based on data from Spain [25]. This could be an underestimate, as the rate from southern China was 3.9% [26]. Finally, we estimated 511 patients with IA annually, or 2.88 cases per 100,000 population. The estimated IA annual incidence is lower than that of Uzbekistan (4.8/100,000) [20], and slightly higher than the Russian Federation (2.27/100,000) and Ukraine (2.7/100,000), although these countries did not include IA after lung cancer in their estimates [5,21].

Chronic pulmonary aspergillosis (CPA) usually affects the patients suffering from chronic pulmonary diseases (tuberculosis, sarcoidosis, COPD, non-tuberculous mycobacterial infection, prior pneumothorax and others). In Kazakhstan in 2015, the total number of patients with pulmonary tuberculosis was 10,296 (58.5/100,000) [6]. The calculation of the possible cases of CPA was made by a formula proposed by Denning and co-authors [12]; 659 new CPA patients following tuberculosis (1.47 cases per 100,000 population). Assuming a 15% annual mortality, the 5 years period prevalence is 2077. Assuming that 33% of CPA cases are attributable to TB, the total CPA prevalence in the Republic is 6231 affected patients (35.5/100,000). This corresponds to Russian Federation and Ukraine data, but in Uzbekistan the prevalence estimate was much lower [5,20,21].

The prevalence of ABPA was estimated among those registered in 2015 with bronchial asthma (12,478 persons) and with CF—68 patients [6]. Therefore, we obtained an estimate of 306 patients with ABPA (1.7/100,000). The estimate of those with SAFS was 1408 in 2015 (2.4/100,000). The prevalence of ABPA and SAFS appear to be similar to Uzbekistan data (2.86 and 3.73 per 100,000 population, respectively) [20], but considerably below those in the Russian Federation (122.5 and 161.7, respectively) [5] and Ukraine (62.5 and 82.3, respectively) [21], probably because asthma is under-diagnosed or not fully registered in Kazakhstan and Uzbekistan [27]. A WHO-designed World Health Survey in 2002–2003 found 1.47% of the adult population to have clinical asthma [27]—about 170,000—which is substantially higher than the official estimate.

### 3.5. Mucormycosis

The mucormycosis incidence in Kazakhstan in 2015 was calculated using data of MHSD about morbidity with AML. Mucormycosis frequency in the patients with AML accounted to 3.6%, and AML was a risk factor for mucormycosis in only 25% of cases [5]. Taking into account that in Kazakhstan in 2015 there were 113 cases of AML, the total number of mucormycosis patients was 16, or 0.09 cases per 100,000 population, which is similar to Uzbekistan data and approximately half the corresponding indices in the Russian Federation and Ukraine [5,20,21].

### 3.6. Pneumocystis Pneumonia and Cryptococcal Meningitis

*Pneumocystis* pneumonia and cryptococcal meningitis seem to be important opportunistic infections in patients with HIV-infection. In Kazakhstan in 2015, there were 17,726 patients living with HIV and 74.6% of them received ART [7]. According to our calculations, there were 1956 patients with *Pneumocystis* pneumonia, or 11.1 cases per 100,000 population. This parameter exceeds data in Uzbekistan (5.37/100,000), but is lower than the parameter in Ukraine (13.5/100,000) [5,20,21]. In European counties, the given parameter was much lower (France—1/100,000, United Kingdom—0.33/100,000) [18].

The total number of cryptococcal meningitis in Kazakhstan in 2015 accounted for 78 cases (0.44/100,000). In Uzbekistan, Russia and Ukraine the incidence of this mycosis appeared to be lower (0.21, 0.21 and 0.22 per 100,000 population) [5,20,21].

## 4. The Limitations of the Study

Owing to the lack of statistics, the number of patients with severe and chronic mycoses in Kazakhstan can only be estimated. Further epidemiological surveys are needed in order to obtain real data about mycoses-related morbidity and mortality in the Republic.

## 5. Conclusions

This is the first attempt to estimate the total burden of fungal disease in the Republic. It shows that mycoses in Kazakhstan, as well as in many other countries, including the Russian Federation, Uzbekistan and Ukraine, are more common than anticipated, but lower than in many other countries, with the exception of ABPA and SAFS. These low rates could reflect lower populations at risk, undiagnosed populations at risk (such as bronchial asthma) and limited fungal diagnostic capacity in Kazakhstan (lack of trained specialists, absence of important tests, for example, *Aspergillus* antigen or *Pneumocystis* PCR etc.). While the azoles (fluconazole, itraconazole and voriconazole), amphotericin B and echinocandins are available in Kazakhstan, flucytosine is not, and no therapeutic antifungal monitoring capability for the azoles is currently available. Establishment of a reference laboratory for medical mycology in Kazakhstan would be the most useful first step in addressing current deficiencies and uncertainties and will contribute towards achieving control of antimicrobial resistance.

## Figures and Tables

**Table 1:** Fungal diseases in the Republic of Kazakhstan (incidence and prevalence estimates for 2015).

Fungal Diseases		Incidence Per 100,000 People	Prevalence
Acute	Invasive aspergillosis	2.8	511
	Invasive candidiasis	4.3	765
	Cryptococcal meningitis	0.44	78
	Mucormycosis	0.09	16
	*Pneumocystis* pneumonia	11.1	1956
	Tinea capitis	66.9	11,847
Subtotal			15,172
Chronic	Recurrent vulvovaginal candidiasis	2985 *	273,258
	Recurrent candidiasis of the oral cavity	24.8	4393
	Recurrent oesophageal candidiasis	5.9	1042
	Chronic pulmonary aspergillosis	35.5	6231
	Allergic bronchopulmonary aspergillosis	1.7	306
	Severe bronchial asthma with fungal sensitization	2.4	422
Subtotal			285,652
Total			300,824

* Incidence per 100,000 females.
